# Lectures on Diseases of the Nervous System

**Published:** 1904-04

**Authors:** 


Lectures on Diseases of the Nervous System. Second series. Subjective Sensations on Light and Sound, Abiotrophy and other Lectures. By Sir William R. Gowers, M. D., F. R. C. P., F. R. S., Honorary Fellow Royal College of Physicians, Ireland, etc. Small octavo, pp. 250. Philadelphia: P. Blakiston's Son & Co. 1904.

The student of medicine, or of any branch thereof, cannot fail to find food for thought in the writings of Gowers. This is because the author has always based his specialism upon the widest knowledge of general medicine. In his lecture on specialism he places himself on record in the following terms:

"Every province of disease abounds in illustrations of the degree to which the special involves the general. No man can be a true specialist who has not an adequate practical acquaintance with the whole range of one of the two great branches of professional knowledge,—surgery, medicine. There is no part of medicine in which a so-called nerve specialist must not be at home. These facts must be recognised by practitioners."

Because of this belief and because Gowers has always written from the standpoint of the neurologist with a practical acquaintance of the whole range of medicine, his lectures are of value to the profession at large. The present series of ten lectures is full of interest and is of special value. J. W. P.

